# Low density lipoprotein and liposome mediated uptake and cytotoxic effect of N^4^-octadecyl-1-**β**-D-arabinofuranosylcytosine in Daudi lymphoma cells

**DOI:** 10.1038/sj.bjc.6690558

**Published:** 1999-07

**Authors:** S K M Koller-Lucae, H Schott, R A Schwendener

**Affiliations:** Department of Pathology, Division of Cancer Research, University Hospital, Schmelzbergstrasse 12, CH-8091 Zurich, Switzerland; Institute of Organic Chemistry, Eberhard-Karls University, Auf der Morgenstelle 18, D-72076 Tübingen, Germany

**Keywords:** ara-C, lipophilic derivative, low density lipoproteins, liposomes, Daudi cells, lymphocytes

## Abstract

Low density lipoprotein (LDL) receptor-mediated uptake and cytotoxic effects of N^4^-octadecyl-1-β-D-arabinofuranosylcytosine (NOAC) were studied in Daudi lymphoma cells. NOAC was either incorporated into LDL or liposomes to compare specific and unspecific uptake mechanisms. Binding of LDL to Daudi cells was not altered after NOAC incorporation (*K*_D_ 60 nM). Binding of liposomal NOAC was not saturable with increasing concentrations. Specific binding of NOAC-LDL to Daudi cells was five times higher than to human lymphocytes. LDL receptor binding could be blocked and up- or down-regulated. Co-incubation with colchicine reduced NOAC-LDL uptake by 36%. These results suggested that NOAC-LDL is taken up via the LDL receptor pathway. In an in vitro cytotoxicity test, the IC_50_ of NOAC-LDL was about 160 μM, whereas with liposomal NOAC the IC_50_ was 40 μM. Blocking the LDL receptors with empty LDL protected 50% of the cells from NOAC cytotoxicity. The cellular distribution of NOAC-LDL or NOAC-liposomes differed only in the membrane and nuclei fraction with 13% and 6% respectively. Although it is more convenient to prepare NOAC-liposomes as compared to the loading of LDL particles with the drug, the receptor-mediated uptake of NOAC-LDL provides an interesting rationale for the specific delivery of the drug to tumours that express elevated numbers of LDL receptors. © 1999 Cancer Research Campaign


*British Journal of Cancer* (1999) **80**(10), 1542–1549
© 1999 Cancer Research Campaign
Article no. bjoc.1999.0558

### Low density lipoprotein and liposome mediated uptake and cytotoxic effect of N⁴-octadecyl-1-β-D-arabinofuranosylcytosine in Daudi lymphoma cells

**SKM Koller-Lucae[1], H Schott[2] and RA Schwendener[1]**

[1]Department of Pathology, Division of Cancer Research, University Hospital, Schmelzbergstrasse 12, CH-8091 Zurich, Switzerland; [2]Institute of Organic Chemistry, Eberhard-Karls University, Auf der Morgenstelle 18, D-72076 Tübingen, Germany

**Summary** Low density lipoprotein (LDL) receptor-mediated uptake and cytotoxic effects of $N^4$-octadecyl-1-β-D-arabinofuranosylcytosine (NOAC) were studied in Daudi lymphoma cells. NOAC was either incorporated into LDL or liposomes to compare specific and unspecific uptake mechanisms. Binding of LDL to Daudi cells was not altered after NOAC incorporation ($K_D$ 60 nM). Binding of liposomal NOAC was not saturable with increasing concentrations. Specific binding of NOAC-LDL to Daudi cells was five times higher than to human lymphocytes. LDL receptor binding could be blocked and up- or down-regulated. Co-incubation with colchicine reduced NOAC-LDL uptake by 36%. These results suggested that NOAC-LDL is taken up via the LDL receptor pathway. In an in vitro cytotoxicity test, the $IC_{50}$ of NOAC-LDL was about 160 μM, whereas with liposomal NOAC the $IC_{50}$ was 40 μM. Blocking the LDL receptors with empty LDL protected 50% of the cells from NOAC cytotoxicity. The cellular distribution of NOAC-LDL or NOAC-liposomes differed only in the membrane and nuclei fraction with 13% and 6% respectively. Although it is more convenient to prepare NOAC-liposomes as compared to the loading of LDL particles with the drug, the receptor-mediated uptake of NOAC-LDL provides an interesting rationale for the specific delivery of the drug to tumours that express elevated numbers of LDL receptors.

**Keywords:** ara-C; lipophilic derivative; low density lipoproteins; liposomes; Daudi cells; lymphocytes

$N^4$-octadecyl-1-β-D-arabinofuranosylcytosine, NOAC (Schott et al, 1994) is a good candidate for cancer therapy with potential advantages compared to the related drug 1-β-D-arabinofuranosylcytosine (ara-C) and to other lipophilic ara-C derivatives. To overcome the disadvantage of rapid deamination of ara-C to the biologically inactive metabolite 1-β-D-arabinofuranosyluracil (ara-U), a large number of chemical modifications of ara-C were made in the past. We synthesized lipophilic ara-C derivatives with $C_{16}$–$C_{22}$ $N^4$-alkyl side chains of which NOAC ($C_{18}$) has the highest activity in murine leukaemias and solid tumour xenograft models (Schwendener et al, 1995). Due to the lipophilic property of NOAC, the drug has to be incorporated in liposomes to obtain a formulation that can be used for intravenous applications. The pharmacological properties of the alkyl-ara-C-derivatives are different from ara-C regarding cellular uptake, formation of ara-C-5′-triphosphate and induction of apoptosis (Horber et al, 1995*a*, 1995*b*, 1995*c*). An important finding was, that NOAC is not tightly anchored to the liposomal lipid bilayer and thus is readily distributed in the blood mainly into erythrocyte membranes and lipoproteins (Koller-Lucae et al, 1997). It is known that unilamellar liposomes aggregate with low density lipoproteins (LDL) and allow the transfer of incorporated drugs from the liposomes to LDL. Similar to the transfer of liposomal phospholipid-ara-C conjugates to LDL as described by MacCoss et al (1983), we also observed a strong affinity of NOAC for lipoproteins. The incubation of liposomal NOAC with human serum resulted in binding to lipoproteins of 36% to LDL and 21% to high density lipoproteins (HDL) (Koller-Lucae et al, 1997). Thus, liposomes provide an ideal formulation for NOAC to assure the transfer of the drug to lipoproteins, especially LDL.

Similar results were reported by Wasan and co-workers (1997) for liposomal nystatin who found an increased distribution into lipoproteins compared to free drug. The natural affinity of NOAC for LDL provides an interesting rationale for the specific delivery of the drug to tumours. Growing and dividing cells require cholesterol for membrane synthesis which is delivered by LDL (Goldstein et al, 1983). This accounts also for cancer cells where an increased LDL uptake in tumours with high metastatic potential and aggressive or undifferentiated character was found (Firestone, 1994). The elevated number of LDL receptors expressed on tumour cells has been exploited for the delivery of lipophilic drugs. Allison et al (1994) described the tumour targetting of photosensitizers using LDL as drug carriers and van Berkel et al (1996) used a lipid emulsion mimicking lipoprotein particles loaded with a lipophilic antiviral drug for increased liver uptake.

Compared to normal blood cells, the LDL receptors of lymphoma and leukaemia cells are up-regulated leading to an increased uptake of LDL (Yen et al, 1994). For our investigation we used the Daudi Burkitt lymphoma cell line, whose LDL binding and uptake properties were characterized by Yen et al (1995). For this in vitro model system we incorporated NOAC into LDL and investigated the uptake mechanisms, cytotoxic activity and cellular distribution of NOAC-LDL in comparison to liposomal NOAC.

*Received 19 October 1998*
*Revised 5 February 1999*
*Accepted 11 February 1999*

*Correspondence to:* RA Schwendener

### MATERIALS AND METHODS

### Chemicals

NOAC was synthesized as described before (Schott et al, 1994) and obtained from Spagomed AG (Burgdorf, Switzerland). Soy phosphatidylcholine (SPC) was obtained from L Meyer (Hamburg, Germany). Cholesterol (Fluka AG, Buchs, Switzerland) was recrystalized from methanol. A stock solution of the fluorescent probe DiI (1,1′-dioctadecyl-3,3,3′,3′-tetramethyl-indocarbocyanine perchlorate, Molecular Probes, Leiden, The Netherlands) was prepared by dissolving 1 mg in 1 ml ethanol for the labelling of liposomal preparations and 2.5 mg in 1 ml dimethyl sulphoxide (DMSO) for LDL. DL-α-Tocopherol, all buffer salts and other chemicals used were of analytical grade and obtained from Merck (Darmstadt, Germany) or Fluka. Soluene 350 and Ultima Gold scintillation cocktail were from Packard Instruments (Groningen, The Netherlands). NOAC was tritium labelled (0.370 GBq $mmol^{-1}$ [5-$^3$H]-NOAC) by Amersham (Amersham, UK). Ara-C (1-β-D-arabinofuranosylcytosine) was dissolved in saline containing 0.01% EDTA (pH 7.4; saline–EDTA) to 10 mM stock solution.

### Culture of Daudi cells and isolation of lymphocytes

The human Burkitt lymphoma cell line, Daudi was obtained from Dr K Melief (The Netherlands Cancer Institute, Amsterdam, The Netherlands). The cells were grown in RPMI-1640 medium (Gibco, Paisley, UK) supplemented with 10% heat-inactivated fetal calf serum (FCS; Gibco), 100 units $ml^{-1}$ penicillin-streptomycin (Gibco) and 2 mM L-glutamine (Gibco) in a humidified 5% carbon dioxide atmosphere. Cells were subcultured three times a week at a split ratio of 1:3 to maintain a density of 0.5–2.0 × $10^6$ cells $ml^{-1}$. Lymphocytes were isolated from fresh human blood ($10^6$ $ml^{-1}$ blood) which was drawn directly into Vacutainer® CPT™ cell preparation tubes (Becton Dickinson, Basel, Switzerland). The tubes were centrifuged (30 min, 1800 ***g***) and the fraction containing the lymphocytes suspended in 15 ml medium supplemented with 10% FCS, centrifuged (10 min, 300 ***g***) and cultured in the same medium over night. On the next day the cells were transferred to new culture flasks to remove monocytes. The homogeneity of the lymphocyte population was analysed in a flow cytometer (Coulter Epics Elite, Miami, FL, USA) by staining the cells with fluorescein isothiocyanate (FITC)-conjugated anti-CD45 and PE-conjugated anti-CD14 antibodies (Coulter), yielding lymphocyte populations of >92%. For the up- or down-regulation of the LDL receptors the Daudi cells or lymphocytes were either cultured for 48 h in serum-free medium (RPMI-1640, penicillin–streptomycin 100 units $ml^{-1}$, L-glutamine 2 mM) supplemented with 10% human lipoprotein-deficient serum (LPDS, Sigma, Buchs, Switzerland) or by replacement of LPDS with 10% FCS supplemented with additional human LDL at a final concentration of 440 nM LDL.

### Preparation of liposomes

Small (100 nm) unilamellar liposomes were prepared in saline–EDTA by sequential filter extrusion as described before (Koller-Lucae et al, 1997). Liposome size and homogeneity was determined by laser light scattering (Submicron Particle Sizer Model 370, Nicomp, Santa Barbara, CA, USA). The basic composition of the liposomes used was 100 mM SPC, 20 mM cholesterol, 0.9 mM DL-α-tocopherol, and 1 mM NOAC, which was trace labelled with [5-$^3$H]-NOAC. Liposomes carrying an average of 100–120 molecules NOAC, as calculated from the liposome diameter and the NOAC concentration, were obtained. For the cytotoxicity experiments liposomes with the same lipid composition were used but NOAC was added at 20 mM (1500 molecules NOAC per liposome). For the flow cytometry experiments liposomes with the same lipid composition, 1 mM DiI and 180 mM sodium cholate were prepared by dialysis against saline–EDTA as described by Rubas et al (1986). DiI concentration was determined with a Spectrofluorometer SFM 23 (Kontron, Zurich, Switzerland) after solubilization of an aliquot of liposomes (20 µl) with 2 ml sodium cholate (0.05%), yielding an average of 80 DiI probes per liposome. For control experiments liposomes without drug or DiI were processed accordingly. All liposomes were sterile filtrated (0.2 µm, Schleicher & Schuell, Feldbach, Switzerland) stored at 4°C and used within 2 weeks.

### Isolation and loading of LDL with DiI or $^3$H-NOAC

LDL was obtained from fresh human plasma by sequential flotation ultracentrifugation (Viallard et al, 1990). Isolated LDL fractions were run on Sephadex G-25 columns (16 × 60 mm, Pharmacia, Uppsala, Sweden) for buffer exchange. In order to prevent oxidation of apolipoprotein $B_{100}$, all media contained 0.01% EDTA. The apolipoprotein $B_{100}$ content of the LDL preparations was measured with a Bio-Rad protein assay (Bio-Rad, Glattbrugg, Switzerland) using albumin as standard. LDL labelling with DiI was performed as described by Morrison et al (1994). DiI concentration was determined fluorometrically as described for DiI-liposomes yielding an average of 100 DiI molecules per LDL. DiI-labelled LDL (DiI-LDL) is still functional in binding to the LDL receptor (Stephan and Yurachek, 1993). The LDL particles were loaded with NOAC as described above for DiI-LDL by incubating 1 mg protein (0.12 ml LDL) with 0.2 mg NOAC (in 0.6 ml DMSO), trace labelled with [5-$^3$H]-NOAC and named $^3$H-NOAC-LDL. This resulted in 40–160 molecules NOAC per LDL which represents an incorporation rate of 20–80%. For the cellular distribution experiment the incorporation of NOAC was 30 molecules NOAC per LDL because the $^3$H-NOAC concentration was increased. The purity of the LDL preparations was assessed on agarose gels as described before (Koller-Lucae et al, 1997). All LDL preparations were sterile filtrated (0.45 µm, Schleicher & Schuell) stored at 4°C and used within 3 weeks. The molarity of the LDL preparations was calculated from the protein concentrations taking into account that the protein content of an LDL particle is 20% of the mass and the molecular mass of LDL is 2500 kDa.

### Binding and association of LDL or liposomes to Daudi cells or lymphocytes

After culturing the cells in medium with 10% FCS, 10% LPDS or 10% FCS containing 440 nM LDL they were washed once with serum-free medium (10 min, 800 ***g***). After culture in LPDS for 48 h the cell viability was judged by trypan blue exclusion resulting in >95% viability for Daudi cells and >90% for lymphocytes respectively. Incubations were performed with 5 × $10^6$ cells $ml^{-1}$ in serum-free medium. For incubations at 4°C the cells were adjusted to temperature for 15 min, before adding liposomes or LDL. Time

© 1999 Cancer Research Campaign

(0.25–24 h) with 144 nM LDL or liposomes and concentration-dependent incubations were performed with $^3$H-NOAC-LDL or $^3$H-NOAC-liposomes (7–280 nM) for 2 h on an overhead shaker (Heidolph, Kelheim, Germany) at 4°C. Each LDL particle and liposome carried an average of 100–120 molecules NOAC.

For binding studies the cells were pre-incubated with saline–EDTA, native LDL or liposomes (250 nM) at 4 or 37°C for 1 h as described above. Then 10 nM $^3$H-NOAC-LDL (60 NOAC molecules per LDL particle) or $^3$H-NOAC-liposomes (130 NOAC molecules per liposome) were added and the incubation continued for 2 h. In some experiments colchicine (1.7 mg ml$^{-1}$) was added to the incubations at 37°C to prevent non-specific uptake by fluid-phase pinocytosis (Swanson et al, 1987). The samples were placed on ice after incubation and washed twice with ice-cold phosphate-buffered saline containing 0.1% bovine serum albumin (PBS–BSA, 10 min, 280 ***g***, 4°C). Supernatant and wash buffers were analysed directly, whereas cell pellets were lysed with 0.2 ml potassium hydroxide (0.2 M) before scintillation counting in a Tri-Carb™ Liquid Scintillation Analyzer (Packard Instruments, Groningen, The Netherlands). All incubations were performed in triplicate. Results were calculated as LDL particles or liposomes bound per cell and binding characteristics were determined by fitting the data with one-site binding hyperbolas.

### Flow cytometry

Time (0.25–4 h with 32 nM LDL or liposomes) and concentration (8–140 nM LDL or liposomes, 2 h) dependent incubations were performed at 4°C with 2.5 × 10$^5$ cells and DiI-LDL or DiI-liposomes, as described above. The cell pellet was suspended in 1 ml PBS–BSA containing 4% formaldehyde. Flow cytometric analysis was performed with an Epics Elite Analyzer (Coulter). DiI-binding or uptake was expressed as mean intensity of fluorescence (MIF) per cell. The experiments were performed in triplicate.

### Cytotoxicity test

Daudi cells were cultured in medium with 10% FCS. Before plating the cells they were washed once in serum-deficient medium. Incubations were performed in flat-bottom 96-well test plates (Techno Plastic Products, Trasadingen, Switzerland) with 0.25 × 10$^6$ cells ml$^{-1}$. NOAC was used at different concentrations ranging from 12.5 to 200 µM. NOAC-LDL (0.13–4 µM LDL), NOAC-liposomes (0.01–0.13 µM liposomes), NOAC dissolved in ethanol (0.13–2 µl ethanol) and ara-C dissolved in saline–EDTA (0.13–2 µl buffer) as well as the carriers without drug were added to the cells. To investigate if excess LDL can block NOAC-LDL activity, the cells were pre-incubated for 25 min with 0.4 and 1.2 µM LDL or the same volume of saline–EDTA. Then 0.6 µM NOAC-LDL corresponding to 100 µM NOAC was added. Incubations were performed at 37°C in a humidified 5% carbon dioxide atmosphere for 24 h. Cell viability of untreated control was 95% as determined by trypan blue exclusion. Then 0.01 ml WST-1 reagent (Boehringer Mannheim, Rotkreuz, Switzerland) was added and the plates were kept in the incubator for 2 h before measuring the absorption on a MRX microplate reader (Dynex Technologies, Chantilly, VA, USA). Results were calculated as per cent of surviving cells by comparison with untreated cells. The 50% inhibitory NOAC concentration (IC$_{50}$) of the different incubations was determined. To compare the effect of serum, the same concentration-dependent incubations were performed with medium containing 5% FCS. All experiments were done in triplicate.

### Cellular distribution of $^3$H-NOAC-LDL and $^3$H-NOAC-liposomes

The experiments were performed as described by Horber (1995*b*). Incubation was performed in 6-well plates (Techno Plastic) with 10$^7$ cells ml$^{-1}$ either with 40 µl $^3$H-NOAC-LDL resulting in 2 µM NOAC (70 nM LDL with 30 molecules NOAC per LDL) or $^3$H-NOAC-liposomes resulting in 200 µM NOAC (3 µM liposomes with 70 molecules NOAC per liposome). Samples were collected after 3 h and total radioactivity in the cell pellet was determined. The cells were lysed by nitrogen gas cavitation and centrifuged to obtain cell fractions. The drug concentration in each fraction was determined by scintillation counting. All experiments were performed in triplicate.

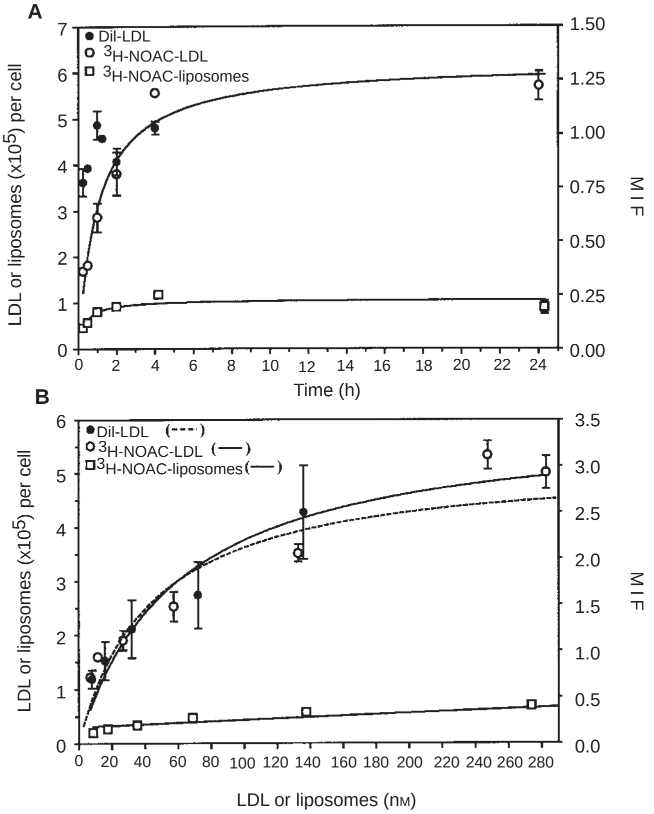

**Figure 1** Time-dependent binding to Daudi cells (**A**) with 144 nM $^3$H-NOAC-LDL (100 NOAC molecules per LDL), 32 nM DiI-LDL (100 DiI probes per LDL) or 144 nM $^3$H-NOAC-liposomes (100 NOAC molecules per liposome). (**B**) Concentration-dependent binding of $^3$H-NOAC-LDL, DiI-LDL or $^3$H-liposomes for 2 h. Binding of LDL particles or liposomes was calculated per cell. DiI-LDL binding was determined by flow cytometry and the results calculated as the mean intensity of fluorescence per cell (MIF). Incubations were performed at 4°C. The results represent means of three experiments. Data were fitted with one-site binding hyperbolas resulting in correlation coefficients of $r$ = 0.948 for NOAC-LDL (**A**), of $r$ = 0.869 for NOAC-liposomes (**A**), of $r$ = 0.934 for NOAC-LDL (**B**) and $r$ = 0.879 for DiI-LDL (**B**). Concentration dependent binding of NOAC-liposomes was fitted by linear regression with $r$ = 0.986 (**B**)

*© 1999 Cancer Research Campaign*

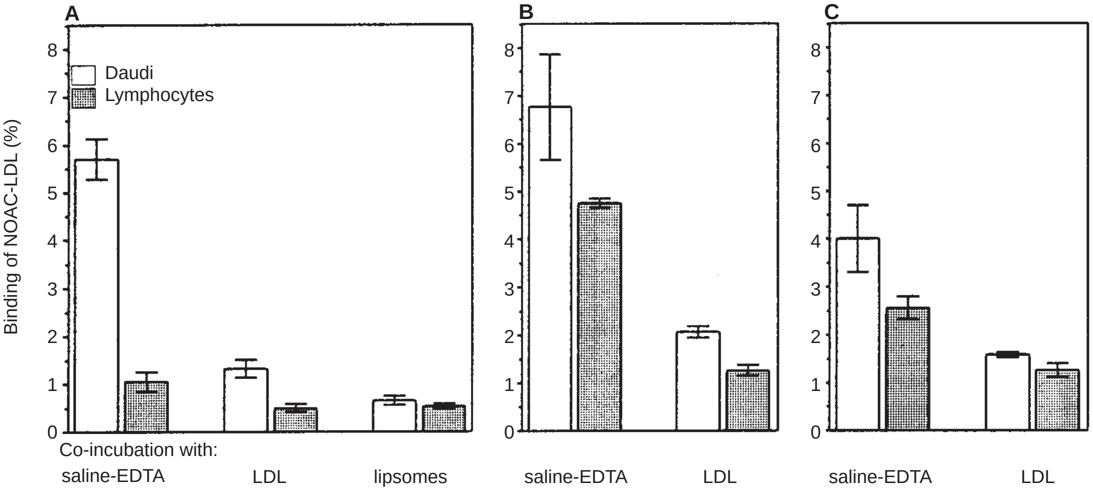

**Figure 2** Effects of culture conditions on the binding of NOAC-LDL to Daudi cells or lymphocytes. The cells were cultured for 48 h in medium with 10% FCS (**A**: native receptors), 10% LPDS (**B**: up-regulated receptors) or 10% FCS plus 440 nM human LDL (**C**: down-regulated receptors) before incubation with NOAC-LDL (10 nM NOAC) for 2 h at 4°C with (LDL, liposomes) or without (saline–EDTA) competitors, which were added 1 h before NOAC-LDL. Binding is shown as % of total drug added

### RESULTS

### Time- and concentration-dependent binding of NOAC

Binding studies were carried out at 4°C in order to minimize internalization processes. Incubation of Daudi cells with $^3$H-NOAC-LDL (144 nM LDL) during 0.25–24 h (Figure 1A) showed that after 2 h saturation of binding was reached. Incubation with different $^3$H-NOAC-LDL concentrations (Figure 1B) for 2 h resulted in a high binding affinity to the LDL receptors with a $K_D$ of 60 nM, a value which was also reported by Yen et al (1995) for LDL binding to Daudi cells. Maximal binding for time or concentration-dependent experiments was 600 000 LDL particles per cell. Incubations with DiI-LDL (Figure 1B) resulted in a similar $K_D$ of 44 nM. Time-dependent incubations with DiI-LDL (Figure 1A, closed circles) were not performed long enough to calculate saturation but data correlate with $^3$H-NOAC-LDL binding. $^3$H-NOAC-liposome binding was investigated under the same conditions. Binding was complete after 40 min with 144 nM liposomes resulting in 100 000 liposomes per cell. However, with increasing concentrations of $^3$H-NOAC-liposomes no saturation was reached, resulting in a slight linear increase of binding up to 280 nM (Figure 1B). Incubations with DiI-liposomes also showed maximal binding after 32 min and no saturation with increasing DiI-liposome concentrations (data not shown).

### Binding of NOAC-LDL to Daudi cells and lymphocytes at 4°C

Incubating Daudi cells which were previously kept during 48 h in medium containing 10% FCS with $^3$H-NOAC-LDL (10 nM LDL) for 2 h at 4°C resulted in a fivefold increased binding compared to lymphocytes (Figure 2A, left bars) reflecting the higher number of LDL-receptors on Daudi cells. Pre-incubation of the cells with 250 nM LDL (1 h, 4°C) before addition of $^3$H-NOAC-LDL blocked NOAC-LDL binding by 77 ± 6% to Daudi cells and by 52 ± 14% to lymphocytes (Figure 2A, middle bars). Repeating the same experiment with 250 nM empty control liposomes resulted in the blocking of 88 ± 3% and 49 ± 8% of binding to Daudi cells or lymphocytes respectively (Figure 2A, right bars). Similar results were obtained for incubations with DiI-LDL. DiI-LDL binding to Daudi cells was threefold higher than to lymphocytes. The binding of DiI-LDL to Daudi cells was reduced to 14% after pre-incubation with a 25-fold excess of control LDL and to 29% after incubation with excess control liposomes (data not shown).

**Table 1** Binding and uptake of $^3$H-NOAC incorporated in LDL or liposomes to Daudi cells or lymphocytes at 37°C

| Pre-incubation for 1 h | Buffer | | LDL | | Liposomes | |
|---|---|---|---|---|---|---|
| Co-incubation for 2 h | Buffer | Colchicine | Buffer | Colchicine | Buffer | Colchicine |
| | Binding and uptake (%) | | | | | |
| $^3$H-NOAC-LDL | | | | | | |
| Daudi cells | 32.3 ± 1.0 (100%) | 20.6 ± 1.6 (64%) | 15.7 ± 0.1 (49%) | 9.9 ± 0.9 (31%) | 0.9 ± 0.08 (3%) | ND |
| Lymphocytes | 4.8 ± 0.4 (100%) | ND | 1.6 ± 0.1 (33%) | ND | 0.5 ± 0.1 (10%) | ND |
| $^3$H-NOAC-liposomes | | | | | | |
| Daudi cells | 6.0 ± 0.3 (100%) | 4.8 ± 0.2 (80%) | 9.5 ± 0.1 (158%) | ND | 0.6 ± 0.03 (10%) | 0.6 ± 0.05 (10%) |
| Lymphocytes | 0.6 ± 0.1 (100%) | ND | 1.6 ± 0.1 (267%) | ND | 0.2 ± 0.03 (33%) | ND |

Results are means ± s.d. of three experiments; ND, not determined. Binding and uptake was calculated as % of total drug added. The corresponding effects of the different incubation conditions were calculated by taking the standard buffer incubations as 100% (values shown in brackets).

**Table 2** Uptake and cellular distribution of NOAC incorporated in LDL or liposomes after 3 h incubation at 37°C

| | | $^3$H-NOAC-LDL | $^3$H-NOAC-liposomes |
|---|---|---|---|
| **Total uptake** | **(pmol/10$^6$ Daudi cells)** | **105.7 ± 1.7** | **1753.2 ± 64.6** |
| | **(% of total dpm added)** | **54.5 ± 0.9** | **10.1 ±0.4** |
| | | **(% of dpm in cells)** | |
| Nuclei/membranes | | 13.3 ± 1.8 | 5.5 ± 0.5 |
| Mitochondria | | 37.2 ± 6.5 | 39.9 ± 6.0 |
| Lysosomes | | 9.6 ± 0.6 | 10.7 ± 1.6 |
| Microsomes | | 5.4 ± 0.1 | 5.9 ± 0.3 |
| Cytosol | | 1.8 ± 0.2 | 3.0 ± 1.3 |
| Recovery | | 67.2 ± 5.9 | 65.0 ± 5.4 |

Results are mean ± s.d, *n* = 3.

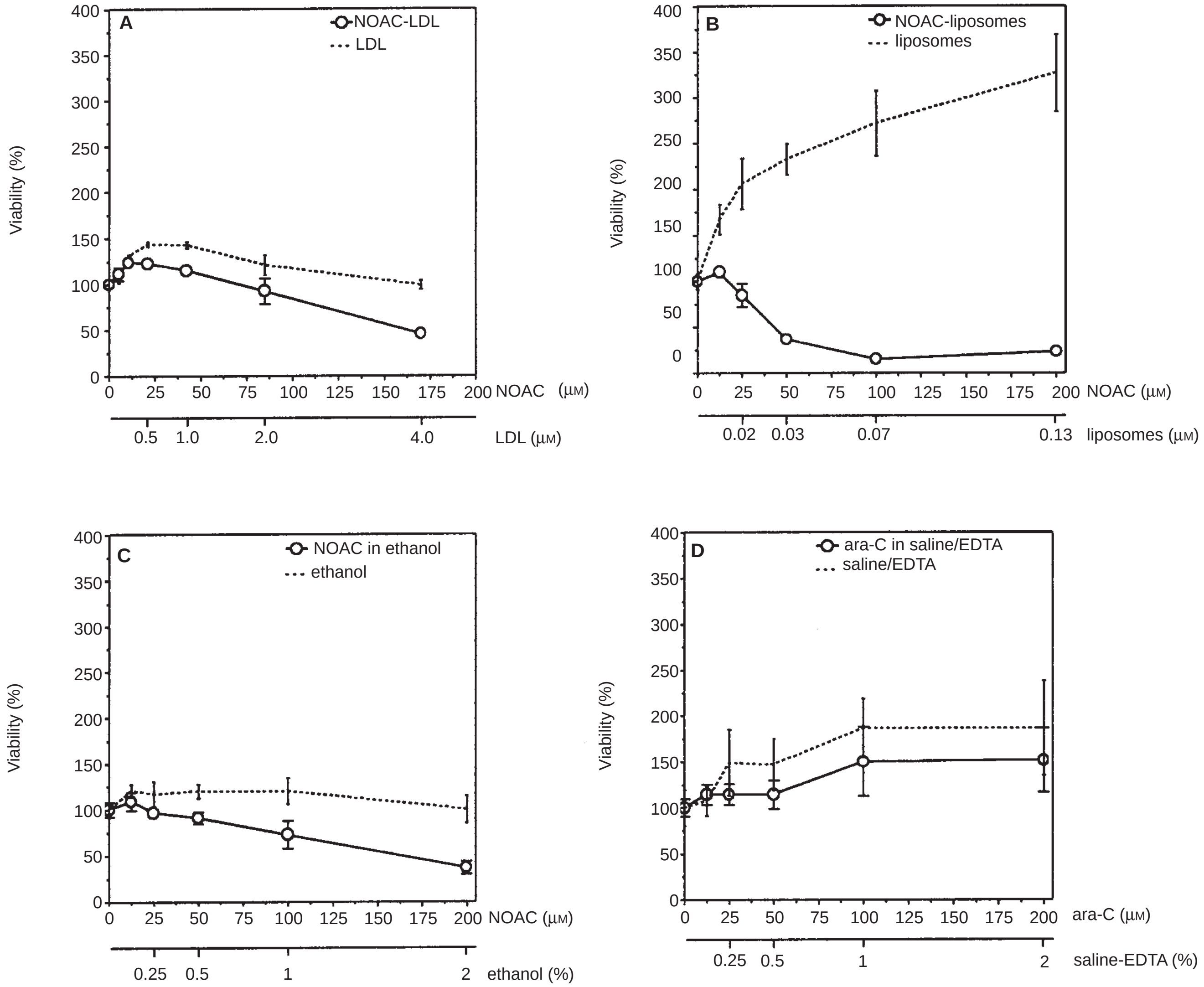

**Figure 3** Cell viability after incubation with different NOAC concentrations incorporated in LDL particles (**A**) or liposomes (**B**) or dissolved in ethanol (**C**) compared to the carriers applied without drug (dotted lines). NOAC and corresponding carrier concentrations are indicated on the x-axes. The effect of ara-C dissolved in saline–EDTA is shown in panel **D**. Incubations were performed during 24 h at 37°C. The results are shown as % viability referred to equal numbers of control cells cultured in incubation medium without FCS

Culture of the cells for 48 h in medium containing 10% LPDS instead of FCS resulted in a fivefold increased binding of $^3$H-NOAC-LDL to lymphocytes and only an insignificant receptor up-regulation on Daudi cells (Figure 2B, left bars). About the same amount of $^3$H-NOAC-LDL binding to up-regulated receptors was blocked with excess control LDL, namely 69 ± 3% on Daudi cells and 74 ± 4% on lymphocytes respectively (Figure 2B, right bars). The up-regulation of LDL-receptors on Daudi cells cultured in

*© 1999 Cancer Research Campaign*

medium containing LPDS was confirmed by flow cytometric investigations using DiI-LDL where a 3-fold shift of the mean intensity of fluorescence (MIF) was observed (data not shown).

Culture of the cells in medium containing 10% FCS and additional human LDL resulted in a 30% decrease of NOAC-LDL binding to Daudi cells but in no reduction for binding to lymphocytes (Figure 2C, left bars). Blocking with excess control LDL reduced the binding only by 60 ± 2% for Daudi cells, whereas for lymphocytes NOAC-LDL binding was reduced by 51 ± 10% (Figure 2C, right bars). The comparison of NOAC-LDL binding to native receptors on lymphocytes (Figure 2A, filled bars) with down-regulated receptors (Figure 2C, filled bars) resulted in the same blocking efficiency of 50%.

### Binding and internalization of NOAC-LDL and NOAC-liposomes at 37°C

Incubations at 37°C resulted in increased binding and internalization of $^{3}$H-NOAC-LDL and $^{3}$H-NOAC-liposomes which was 6–7 times higher in Daudi cells and 3–4 times in lymphocytes as compared to the incubations at 4°C. From the total uptake values shown in Table 1 the corresponding effects of the different incubation conditions were calculated by taking the standard incubations as 100%. Blocking of the LDL receptors with a 25-fold excess of LDL reduced the $^{3}$H-NOAC-LDL uptake by 49% for Daudi cells and by 67% for lymphocytes. After blocking the internalization by fluid phase pinocytosis by co-incubation with colchicine 64% of LDL-mediated NOAC uptake and 80% of liposomal NOAC uptake remained. After blocking with the combination of empty control LDL and colchicine, 31% of $^{3}$H-NOAC-LDL were still taken up by Daudi cells (Table 1). This result indicates that at least these 31% of $^{3}$H-NOAC might have been taken up through the membranes by other mechanisms than receptor-mediated internalization or pinocytosis. Pre-incubation of the cells with 250 nM control liposomes reduced binding of $^{3}$H-NOAC-LDL to 3% and of $^{3}$H-NOAC-liposomes to 10% to Daudi cells and to 10% and 33% to lymphocytes respectively. Interestingly, the incubation of $^{3}$H-NOAC-liposomes with excess LDL increased the $^{3}$H-NOAC binding to Daudi cells by 158% and to lymphocytes by 267%.

### Cytotoxicity

Incubation of Daudi cells with NOAC in LDL for 24 h resulted in a $IC_{50}$ of 160 μM NOAC (Figure 3A). Performing the same experiment with NOAC in liposomes (Figure 3B) or NOAC dissolved in ethanol (Figure 3C) gave $IC_{50}$ values of 40 μM and 150 μM NOAC respectively. The comparison of these incubations with control incubations of identical LDL, liposome or ethanol concentrations without drug revealed a moderate activation of the cell proliferation by LDL, a strong activation by liposomes and no change after incubation with up to 2% (v/v) of ethanol (dotted lines in Figure 3). Ara-C had no cytotoxic effect under these incubation conditions (Figure 3D). Blocking of the LDL receptors with 0.4 μM LDL 25 min before addition of NOAC-LDL (0.6 μM LDL) corresponding to 100 μM NOAC reduced the cytotoxicity by 34% and the blocking of the LDL receptors with a twofold higher LDL concentration of 1.2 μM reduced NOAC cytotoxicity by 51% (data not shown).

To investigate the effect of serum proteins present during the cytotoxicity experiments, 5% FCS was added to the medium. The activating effect of additional lipids and cholesterol provided by liposomes or LDL was less pronounced in the presence of serum proteins. The NOAC-LDL mediated cytotoxicity was not influenced by serum proteins, whereas the cytotoxicity of NOAC-liposomes decreased by a factor of 2 and for NOAC dissolved in ethanol it increased by a factor 2 at a NOAC concentration of 50 μM. According to serum-free incubations, ara-C cytotoxicity was not observed in the presence of FCS.

### Cellular distribution of $^{3}$H-NOAC-LDL or $^{3}$H-NOAC-liposomes

The incubation of Daudi cells for 3 h with 0.07 μM $^{3}$H-NOAC-LDL (30 molecules NOAC per LDL particle) or with 3 μM $^{3}$H-NOAC-liposomes (70 molecules NOAC per liposome) left 55% of $^{3}$H-NOAC-LDL and 10% of the liposomal formulation associated with the cell pellet. Data of cellular drug distribution are presented in Table 2.

The only difference was found in the membrane and nuclei fraction where 13% of the $^{3}$H-NOAC-LDL and 6% $^{3}$H-NOAC-liposomes were found. Highest drug concentrations were found in the mitochondrial fraction for both applications.

### DISCUSSION

In this study we compared cellular uptake and cytotoxicity in Daudi cells of NOAC-LDL to liposomal NOAC. The incorporation of anticancer therapeutics into isolated LDL or LDL-fragments for tumour targeting was investigated by several groups. Rensen et al (1997) prepared LDL-like particles by using liposomes which were modified with human recombinant apolipoprotein E. They found a LDL receptor-mediated uptake for these modified liposomes to B16 melanoma cells, demonstrating that these liposomes could be used to carry antineoplastic drugs to tumours. Vitols et al (1990) incorporated a water-insoluble alkylating agent into LDL by incubating lyophilized LDL with the drug solubilized in heptane followed by solvent evaporation before adding buffer to the LDL–drug complex. Such elaborate in vitro preparations are not necessary for NOAC because earlier investigations revealed that NOAC is spontaneously transferred from liposomes to LDL in human blood (Koller-Lucae et al, 1997).

The binding properties of LDL to the LDL receptor were not impaired after incorporation of NOAC because comparable association constants were found for NOAC-LDL and DiI-LDL. This is consistent with findings for other lipophilic compounds like indium complexed to DTPA-stearylamide, which did not hinder LDL-specific interactions with cells after incorporation into the lipophilic part of the LDL particles (Jasanada et al, 1996). Yen and co-workers (1995) reported that LDL receptors on Daudi cells were already up-regulated which was reflected by elevated mRNA for LDL receptors and insignificant increase of receptor density after incubation in lipoprotein-deficient medium. Under normal culture conditions LDL receptor expression on lymphocytes was low which resulted in receptor up-regulation in lipoprotein-deficient medium but in no down-regulation in the presence of additional LDL in the culture medium. These results that are valid for LDL interactions with lymphoma cells and lymphocytes were confirmed in this study for $^{3}$H-NOAC incorporated into LDL (Figure 3 A–C). Therefore we conclude that NOAC incorporated into LDL can be taken up by the LDL receptor-mediated route. The LDL receptor-mediated uptake of NOAC was also observed

© 1999 Cancer Research Campaign 

when liposomal NOAC was co-incubated with control LDL. This route of uptake was probably followed by transfer of NOAC molecules from the liposomes to LDL (Table 1).

In addition to specific binding, NOAC-LDL interacted also unspecifically with cells because at 4°C only 77% of the binding could be blocked with excess empty LDL. Goldstein and Brown (1977) found that about 75% of LDL uptake by cells is LDL receptor-mediated and they also observed LDL internalization on fibroblasts by receptor-independent endocytosis. For the LDL-associated cholesterol esters a receptor-independent uptake has been described. After release from LDL these esters interact with plasma membranes and are taken up by cells through an unspecific mechanism (Rinninger et al, 1995). Thus, the co-transfer of cholesterol esters and NOAC from LDL directly into cell membranes could represent another possible mode of uptake for NOAC. This hypothesis is supported by our finding that 31% of NOAC binding and uptake has remained even after blocking receptor-mediated LDL-uptake with excess LDL and co-incubation with colchicine to block unspecific pinocytosis. Accordingly, Vitols et al (1990) described a LDL receptor-independent effect resulting in the cytotoxic activity of a lipophilic mitoclomine-derivative–LDL complex in mutated CHO cells which were deprived of LDL receptors. This is in agreement with our results of the cytotoxicity studies where 50% of the toxic effect of NOAC-LDL remained after blocking the LDL receptors with empty control LDL. Nevertheless, the cytotoxic effect of liposomal NOAC was more pronounced than with NOAC-LDL, resulting in $IC_{50}$ values of 40 μM and 160 μM respectively. NOAC-LDL was shown to bind specifically with a much higher affinity to Daudi cells than liposomal NOAC (Figure 1, Table 1). However, it has to be taken into account that binding and uptake studies were performed with NOAC-LDL and liposomal NOAC where each carrier contained equal amounts of the drug, whereas for the cytotoxicity experiments liposomes carried about 40 times more NOAC than the LDL particles (Figure 3). This means, that even though liposomes bind less efficiently to Daudi cells they take up 40 times more drug with one liposome compared to one LDL particle. Therefore the cytotoxic dose is reached earlier in cells which are incubated with liposomal NOAC. The difference in NOAC accumulation in cells when the carriers are loaded with different amounts of drug is also presented in Table 2 for the different drug carriers used. Additional lipids and cholesterol administered as liposomes or LDL resulted in increased cell proliferation and a viability of more than 100% compared to cells cultured in serum-free medium (Figure 3A, B). The activating effect of additional lipids and cholesterol provided by liposomes or LDL was less pronounced in the presence of serum proteins. The increased toxicity of NOAC dissolved in ethanol in incubations with additional FCS was probably due to stabilization of NOAC in the aqueous incubation medium by serum proteins, preventing crystallization of excess drug. The decreased cytotoxicity of liposomal NOAC under the same conditions may be caused by interfering plasma proteins which bind to the liposomal surface to prevent interaction with cells (Juliano, 1988). Ara-C was not toxic to Daudi cells in incubations with or without FCS. This was already described by Abe and his group (1982), who observed ara-C toxicity only after 7 days of drug incubation. Vogler and colleagues (1991) reported for different cytotoxic alkyl lysophospholipids $IC_{50}$ values determined in Daudi cells which were comparable to our findings for NOAC.

In summary, the uptake experiments with the Daudi cells which have highly up-regulated LDL receptors, resulted in a more efficient uptake of NOAC-LDL compared to NOAC-liposomes. The NOAC-LDL particles are taken up by the receptor route because binding and internalization as well as toxicity can partially be blocked with excess empty LDL. However, total blocking was not achieved, suggesting the involvement of other unspecific uptake mechanisms. NOAC does not necessarily need to be transferred to LDL in order to act as a cytotoxic drug, since liposomal NOAC was also active (cf. Figure 3B). Liposomes have the advantage that they can carry much more NOAC molecules than LDL. In addition, liposomal drug preparations are much easier to formulate for clinical preparations. The new anticancer drug NOAC has several properties that are different to the parent drug ara-C. This was demonstrated by its anti-tumour activity in the Daudi lymphoma cells in this study, by comparative pharmacokinetic studies and in xenograft mouse models (Schwendener et al, 1995). The transfer of NOAC from liposomes to serum LDL could result in increased drug uptake in tumour cells that express elevated LDL receptor numbers, and thus, lead to an improved anti-tumour effect as compared to other drugs.

### ACKNOWLEDGEMENTS

The authors thank R Cattaneo, C Marty and E Brunner for their assistance. We also gratefully acknowledge E Niederer at the Institute of Medical Engineering of the University and Swiss Federal Institute of Technology, Zurich for the flow cytometry measurements. This work was supported by a grant from the EG, G, G, and Ch Sassella Foundation (to SKMK-L) and in part by a grant from the Stiftung für angewandte Krebsforschung, Stiftung für Krebsforschung and Stiftung zur Krebsbekämpfung Zurich (to RAS).

### REFERENCES

Abe I, Saito S, Hori K, Suzuki M and Sato H (1982) Role of dephosphorylation in accumulation of 1-β-D-arabinofuranosylcytosine-5′-triphosphate in human lymphoblastic cell lines with reference to their drug sensitivity. *Cancer Res* **42**: 2846–2851

Allison BA, Pritchard PH and Levy JG (1994) Evidence for low-density lipoprotein receptor-mediated uptake of benzoporphyrin derivative. *Br J Cancer* **69**: 833–839

Firestone RA (1994) Low-density lipoprotein as a vehicle for targeting antitumor compounds to cancer cells. *Bioconjug Chem* **5**: 105–113

Goldstein JL, Basu SK and Brown MS (1983) Receptor-mediated endocytosis of low-density lipoprotein in cultured cells. *Methods Enzymol* **98**: 241–260

Goldstein JL and Brown MS (1977) The low-density lipoprotein pathway and its relation to atherosclerosis. *Annu Rev Biochem* **46**: 897–930

Horber DH, Schott H and Schwendener RA (1995*a*) Cellular pharmacology of a liposomal preparation of $N^4$-hexadecyl-1-β-D-arabinofuranosylcytosine, a lipophilic derivative of 1-β-D-arabinofuranosylcytosine. *Br J Cancer* **71**: 957–962

Horber DH, Schott H and Schwendener RA (1995*b*) Cellular pharmacology of $N^4$-hexadecyl-1-β-D-arabinofuranosylcytosine in the human leukemic cell lines K-562 and U-937. *Cancer Chemother Pharmacol* **36**: 483–492

Horber DH, von Ballmoos P, Schott H and Schwendener RA (1995*c*) Cell cycle dependent cytotoxicity and induction of apoptosis by liposomal $N^4$-hexadecyl-1-β-D-arabinofuranosylcytosine. *Br J Cancer* **72**: 1067–1073

Jasanada F, Urizzi P, Souchard JP, Le Gaillard F, Favre G and Nepveu F (1996) Indium-111 labeling of low density lipoproteins with the DTPA-bis(stearylamide): evaluation as a potential radiopharmaceutical for tumor localization. *Bioconjug Chem* **7**: 72–81

Juliano RL (1988) Factors affecting the clearance kinetics and tissue distribution of liposomes, microsperes and emulsions. *Adv Drug Deliv Rev* **2**: 31–45

© 1999 Cancer Research Campaign

Koller-Lucae SKM, Schott H and Schwendener RA (1997) Interactions with human blood in vitro and pharmacokinetic properties in mice of liposomal N[4]-octadecyl-1-β-D-arabinofuranosylcytosine, a new anticancer drug. *J Pharmacol Exp Ther* **282**: 1572–1580

Koller-Lucae SKM, Suter MJ-F, Rentsch KM, Schott H and Schwendener RA (1999) Metabolism of the new liposomal anticancer drug N[4]-octadecyl-1-β-D-arabinofuranosylcytosine (NOAC). *Drug Metabol Dispos* **27**: 342–350

MacCoss M, Edwards JJ, Lagocki P and Rahman YE (1983) Phospholipid-nucleoside conjugates. The interaction of selected 1-β-D-arabinofuranosylcytosine-5′-diphosphate-L-1,2-diacylglycerols with serum lipoproteins. *Biochem Biophys Res Commun* **116**: 368–374

Morrison IE, Anderson CM, Georgiou GN, Stevenson GV and Cherry RJ (1994) Analysis of receptor clustering on cell surfaces by imaging fluorescent particles. *Biophys J* **67**: 1280–1290

Rensen PCN, Schiffelers RM, Versluis AJ, Bijsterbosch MK, Van-Kuijk MM and van Berkel T (1997) Human recombinant apolipoprotein E-enriched liposomes can mimic low-density lipoproteins as carriers for the site-specific delivery of antitumor agents. *Mol Pharmacol* **52**: 445–455

Rinninger F, Brundert M, Jackle S, Kaiser T and Greten H (1995) Selective uptake of low-density lipoprotein-associated cholesteryl esters by human fibroblasts, human HepG2 hepatoma cells and J774 macrophages in culture. *Biochim Biophys Acta* **1255**: 141–153

Rubas W, Supersaxo A, Weder HG, Hartmann HR, Hengartner H, Schott H and Schwendener R (1986) Treatment of murine L1210 lymphoid leukemia and melanoma B16 with lipophilic cytosine arabinoside prodrugs incorporated into unilamellar liposomes. *Int J Cancer* **37**: 146–154

Schott H, Häussler MP and Schwendener RA (1994) Synthese von 4-Alkylcytosinnucleosiden und deren cytostatische Wirkung im L1210 Leukämiemodell der Maus. *Liebigs Ann Chem* 465–470

Schwendener RA, Horber DH, Ottiger C, Rentsch KM, Fiebig HH and Schott H (1995) Alkasar-18,1-(β-D-arabinofuranosyl)-4-octadecylamino-2(1H)-pyrimidin-one, N[4]-octadecyl-ara-C, NOAC. *Drugs of the Future* **20**: 11–15

Stephan ZF and Yurachek EC (1993) Rapid fluorometric assay of LDL receptor activity by DiI-labeled LDL. *J Lipid Res* **34**: 325–330

Swanson J, Bushnell A and Silverstein SC (1987) Tubular lysosome morphology and distribution within macrophages depend on the integrity of cytoplasmic microtubules. *Proc Natl Acad Sci USA* **84**: 1921–1925

van Berkel TJ, van Dijk MCM, Bijsterbosch MK and Rensen PCN (1996) Drug targeting by neo-lipoproteins. *J Controlled Release* **41**: 85–90

Viallard V, Lacombe C, Trocheris V, Tabacik C and Aliau S (1990) Metabolism of low-density lipoprotein in differentiated and undifferentiated HT29 colon cancer cells. *Int J Cancer* **46**: 320–325

Vitols S, Soderberg Reid K, Masquelier M, Sjostrom B and Peterson C (1990) Low density lipoprotein for delivery of a water-insoluble alkylating agent to malignant cells. In vitro and in vivo studies of a drug-lipoprotein complex. *Br J Cancer* **62**: 724–729

Vogler WR, Olson AC, Okamoto S, Shoji RL, Kuo JF, Berdel WE, Eibl H, Hajdu J and Nomura H (1991) Comparison of selective cytotoxicity of alkyl lysophospholipids. *Lipids* **26**: 1418–1423

Wasan KM, Ramaswamy M, Cassidy SM, Kazemi M, Strobel FW and Thies RL (1997) Physical characteristics and lipoprotein distribution of liposomal nystatin in human plasma. *Antimicrob Agents Chemother* **41**: 1871–1875

Yen CF, Kalunta CI, Chen FS, Kaptein JS, Lin CK and Lad PM (1994) Flow cytometric evaluation of LDL receptors using DiI-LDL uptake and its application to B and T lymphocytic cell lines. *J Immunol Methods* **177**: 55–67

Yen CF, Kalunta CI, Chen FS, Kaptein JS, Lin CK and Lad PM (1995) Regulation of low-density lipoprotein receptors and assessment of their functional role in Burkitt's lymphoma cells. *Biochim Biophys Acta* **1257**: 47–57

*© 1999 Cancer Research Campaign*

## References

[PDF_00800] Abe I., Saito S., Hori K., Suzuki M., Sato H. (1982). Role of dephosphorylation in accumulation of 1-beta-D-arabinofuranosylcytosine 5'-triphosphate in human lymphoblastic cell lines with reference to their drug sensitivity.. Cancer Res.

[PDF_00804] Allison B. A., Pritchard P. H., Levy J. G. (1994). Evidence for low-density lipoprotein receptor-mediated uptake of benzoporphyrin derivative.. Br J Cancer.

[PDF_00807] Firestone R. A. (1994). Low-density lipoprotein as a vehicle for targeting antitumor compounds to cancer cells.. Bioconjug Chem.

[PDF_00809] Goldstein J. L., Basu S. K., Brown M. S. (1983). Receptor-mediated endocytosis of low-density lipoprotein in cultured cells.. Methods Enzymol.

[PDF_00811] Goldstein J. L., Brown M. S. (1977). The low-density lipoprotein pathway and its relation to atherosclerosis.. Annu Rev Biochem.

[PDF_00818] Horber D. H., Schott H., Schwendener R. A. (1995). Cellular pharmacology of N4-hexadecyl-1-beta-D-arabinofuranosylcytosine in the human leukemic cell lines K-562 and U-937.. Cancer Chemother Pharmacol.

[PDF_00813] Horber D. H., Schott H., Schwendener R. A. (1995). Cellular pharmacology of a liposomal preparation of N4-hexadecyl-1-beta-D-arabinofuranosylcytosine, a lipophilic derivative of 1-beta-D-arabinofuranosylcytosine.. Br J Cancer.

[PDF_00822] Horber D. H., von Ballmoos P., Schott H., Schwendener R. A. (1995). Cell cycle-dependent cytotoxicity and induction of apoptosis by liposomal N4-hexadecyl-1-beta-D-arabinofuranosylcytosine.. Br J Cancer.

[PDF_00826] Jasanada F., Urizzi P., Souchard J. P., Le Gaillard F., Favre G., Nepveu F. (1996). Indium-111 labeling of low density lipoproteins with the DTPA-bis(stearylamide): evaluation as a potential radiopharmaceutical for tumor localization.. Bioconjug Chem.

[PDF_00834] Koller-Lucae S. K., Schott H., Schwendener R. A. (1997). Interactions with human blood in vitro and pharmacokinetic properties in mice of liposomal N4-octadecyl-1-beta-D-arabinofuranosylcytosine, a new anticancer drug.. J Pharmacol Exp Ther.

[PDF_00839] Koller-Lucae S. K., Suter M. J., Rentsch K. M., Schott H., Schwendener R. A. (1999). Metabolism of the new liposomal anticancer drug N4-octadecyl-1-beta-D-arabinofuranosylcytosine in mice.. Drug Metab Dispos.

[PDF_00843] MacCoss M., Edwards J. J., Lagocki P., Rahman Y. E. (1983). Phospholipid-nucleoside conjugates. 5. The interaction of selected 1-beta-D-arabinofuranosylcytosine-5'-diphosphate-L-1,2-diacylglycerols with serum lipoproteins.. Biochem Biophys Res Commun.

[PDF_00847] Morrison I. E., Anderson C. M., Georgiou G. N., Stevenson G. V., Cherry R. J. (1994). Analysis of receptor clustering on cell surfaces by imaging fluorescent particles.. Biophys J.

[PDF_00850] Rensen P. C., Schiffelers R. M., Versluis A. J., Bijsterbosch M. K., Van Kuijk-Meuwissen M. E., Van Berkel T. J. (1997). Human recombinant apolipoprotein E-enriched liposomes can mimic low-density lipoproteins as carriers for the site-specific delivery of antitumor agents.. Mol Pharmacol.

[PDF_00854] Rinninger F., Brundert M., Jäckle S., Kaiser T., Greten H. (1995). Selective uptake of low-density lipoprotein-associated cholesteryl esters by human fibroblasts, human HepG2 hepatoma cells and J774 macrophages in culture.. Biochim Biophys Acta.

[PDF_00858] Rubas W., Supersaxo A., Weder H. G., Hartmann H. R., Hengartner H., Schott H., Schwendener R. (1986). Treatment of murine L1210 lymphoid leukemia and melanoma B16 with lipophilic cytosine arabinoside prodrugs incorporated into unilamellar liposomes.. Int J Cancer.

[PDF_00869] Stephan Z. F., Yurachek E. C. (1993). Rapid fluorometric assay of LDL receptor activity by DiI-labeled LDL.. J Lipid Res.

[PDF_00871] Swanson J., Bushnell A., Silverstein S. C. (1987). Tubular lysosome morphology and distribution within macrophages depend on the integrity of cytoplasmic microtubules.. Proc Natl Acad Sci U S A.

[PDF_00876] Viallard V., Lacombe C., Trocheris V., Tabacik C., Aliau S. (1990). Metabolism of low-density lipoprotein in differentiated and undifferentiated HT29 colon cancer cells.. Int J Cancer.

[PDF_00879] Vitols S., Söderberg-Reid K., Masquelier M., Sjöström B., Peterson C. (1990). Low density lipoprotein for delivery of a water-insoluble alkylating agent to malignant cells. In vitro and in vivo studies of a drug-lipoprotein complex.. Br J Cancer.

[PDF_00883] Vogler W. R., Olson A. C., Okamoto S., Shoji M., Raynor R. L., Kuo J. F., Berdel W. E., Eibl H., Hajdu J., Nomura H. (1991). Comparison of selective cytotoxicity of alkyl lysophospholipids.. Lipids.

[PDF_00886] Wasan K. M., Ramaswamy M., Cassidy S. M., Kazemi M., Strobel F. W., Thies R. L. (1997). Physical characteristics and lipoprotein distribution of liposomal nystatin in human plasma.. Antimicrob Agents Chemother.

[PDF_00889] Yen C. F., Kalunta C. I., Chen F. S., Kaptein J. S., Lin C. K., Lad P. M. (1994). Flow cytometric evaluation of LDL receptors using DiI-LDL uptake and its application to B and T lymphocytic cell lines.. J Immunol Methods.

[PDF_00892] Yen C. F., Kalunta C. I., Chen F. S., Kaptein J. S., Lin C. K., Lad P. M. (1995). Regulation of low-density lipoprotein receptors and assessment of their functional role in Burkitt's lymphoma cells.. Biochim Biophys Acta.

